# Hollow Tubular Engineering and Electronic Structure Modulation of Vanadium-Incorporated MoP for Boosting Alkaline Hydrogen Evolution

**DOI:** 10.3390/nano16120776

**Published:** 2026-06-19

**Authors:** Wei Yang, Guimin Wang, Siyi Yang, Ganceng Yang, Haijing Yan, Yanqing Jiao

**Affiliations:** Key Laboratory of Functional Inorganic Material Chemistry, National Center for International Research on Catalytic Technology, Heilongjiang University, Ministry of Education of the People’s Republic of China, Harbin 150080, China

**Keywords:** hydrogen evolution reaction, morphology engineering, heteroatom doping, electronic structure modulation, metal phosphides

## Abstract

Synergistically optimizing electronic structure and exposing abundant active sites is a promising route to enhance electrocatalytic activity, yet remains challenging. Herein, a hierarchical tubular structure of vanadium-incorporated molybdenum phosphide (V-MoP) was successfully constructed for highly effectively alkaline hydrogen evolution. Molecular self-assembly of a V-substituted Keggin-type polyoxometalate (POM) with a simple organic ligand was exploited to induce a hollow tubular precursor and trigger precise V doping by virtue of the intrinsic structural features of POMs, thereby realizing simultaneous morphology engineering and electronic structure modulation. The unique open-ended hollow tubular structure, which furnishes both internal and external surfaces and superhydrophilicity, increases the exposure of electrochemical active sites, promotes rapid electrolyte penetration and shortens mass transfer pathways. Moreover, V doping effectively modulates the electronic structure of MoP, further renders Mo and P sites more electron-rich, meanwhile triggering the coexistence of V^3+^ and V^5+^, which further promotes water dissociation and hydrogen evolution. Consequently, the V-MoP catalyst exhibits significantly enhanced activity, far beyond that of pristine bulk MoP and bulk V-MoP, and even surpasses that of commercial 20% Pt/C at high current densities. This work provides a feasible strategy for designing advanced electrocatalysts with tailored morphology and tunable electronic structures.

## 1. Introduction

Hydrogen offers the advantages of high energy density and environmental friendliness, and is an extensively studied promising and clean energy carrier to address future energy crises [1,2,3]. One of the most economical and sustainable methods for producing hydrogen is to combine water electrolysis with renewable energy; this approach represents an efficient and environmentally friendly way to produce high-purity hydrogen [4,5]. Compared to water acidic electrolysis, alkaline water electrolysis demonstrates superior cost-effectiveness and stability in the HER, particularly under high current density conditions, and holds great potential for large-scale application [6,7]. However, because the HER in an alkaline environment requires an additional water dissociation step (Volmer step: H_2_O + e^−^ → H* + OH^−^), its reaction kinetics is approximately two orders of magnitude slower than those under acidic conditions [8,9,10]. Although Pt-based nanomaterials are still considered the optimal materials for the HER, their high cost and scarcity have hindered their widespread application in the hydrogen economy [11,12]. Therefore, the development of non-Pt electrocatalysts capable of efficiently driving the HER at lower overpotentials is of great significance for large-scale hydrogen production.

Transition metal phosphides (TMPs), especially Mo-based phosphides, exhibit good stability, decent electrical conductivity, and Pt-like electronic structures, making them promising candidates to replace Pt-based materials for HER [13,14]. However, Mo-based phosphides usually exhibit unsatisfactory HER activity due to the large size, high density, and slow ionic dynamics caused by high-temperature calcination [15,16]. In this regard, morphology engineering and electron structure modulation have been proven effective in addressing these issue [17,18]. Notably, hollow structures are promising for electrocatalysis, owing to their high specific surface areas for offering abundant active sites, porosity and thin shells for enhancing wettability and fast ion/electron transport [19,20]. Moreover, open-ended hollow structures are more favorable because they further accelerate mass transfer via unobstructed axial channels, leverage both inner and outer walls for catalysis, and avoid gas trapping, thereby boosting activity and durability under high current densities. However, the widespread application remains limited by the complicated and costly hard-template synthesis [21]. In particular, for Mo-based phosphides, developing a template-free method to fabricate open-ended hollow structures is challenging, as the required high temperature treatment inevitably leads to collapse. Moreover, for alkaline HER, pristine Mo-based phosphides suffer from overly strong binding with hydroxy, leading to sluggish Volmer kinetics [22]. Heteroatom doping has been widely applied to regulate electronic structure and optimize the adsorption energy [23]. Encouragingly, owing to its multiple valence states, V doping is highly favorable for regulating the adsorption of various intermediates by bidirectionally adjusting the d-band center of the active sites, as verified in V-CoP_2_/CC and V-Ni_3_N@MoO_x_ [24,25]. Inspired by the above, constructing open-ended hollow tubular structures with V doping can synergistically reduce electrochemical, ohmic, and diffusion polarization to boost HER activity, but achieving both features simultaneously remains a formidable challenge and has rarely been explored.

Herein, we develop a simple POM self-assembly hollowing strategy driven by Ostwald ripening. By employing bimetallic POM clusters (H_5_[PV_2_Mo_10_O_40_]·34H_2_O, denoted as V_2_Mo_10_), an open-ended hollow tubular POM-based precursor was successfully constructed, which was further converted into a V-doped MoP (referred to as V-MoP) hollow tubular structure by controllable phosphorization. Compared to the reported hollow MoP and V-doped strategies, our strategy provides a cost-effective and facile method that avoids hard templates and template removal, offering the distinct advantage of precise V incorporation into the MoP lattice, which benefits from the structural characteristics of the bimetallic polyoxometalate. This approach simultaneously induces the hollow tubes and triggers precise V doping in the MoP lattice, enabling efficient morphology engineering and electronic structure regulation. Structural characterizations indicate that the incorporation of V into the MoP lattice causes lattice contraction and further tunes the electronic structure of MoP, resulting in electron enrichment on Mo and P sites, and the coexistence of V^3+^ and V^5+^. The resulting electronic configuration is expected to synergistically promote water dissociation and intermediate desorption, thereby boosting alkaline HER activity. The open-ended hollow tubular structure is responsible for facilitating the exposure of more catalytic active sites, improving electrolyte wettability, and promoting mass transfer. Consequently, the V-MoP hollow tube catalyst presents an enhanced electrocatalytic HER activity, reducing the overpotential by 44 and 31 mV at 10 mA cm^−2^ and decreasing the Tafel slope by 45% and 19% relative to bulk MoP and bulk V-MoP, respectively. This work provides a simple, inexpensive, and scalable synthetic strategy for designing high-efficiency electrocatalysts.

## 2. Materials and Methods

### 2.1. Materials

Ruthenium (III) chloride hydrate (RuCl_3_·xH_2_O, Aladdin Biochemical Technology Co., Ltd., Shanghai, China, 99.0%), Phosphomolybdic acid, hydrated (H_3_PMo_12_O_40_⋅nH_2_O, Sinopharm Chemical Reagent Co., Ltd., Shanghai, China, 99.0%), 4,4′-bipyridine (Aladdin, 98.0%), Sodium Hypophosphite (NaH_2_PO_2_·H_2_O, Tianjin Kemiou Chemical Reagent Co., Ltd., Tianjin, China, 98.0%), Ethanol (C_2_H_5_OH, Tianjin Fuyu Fine Chemical Co., Ltd., Tianjin, China). Lsopropyl alcohol (C_3_H_8_O, Tianjin Fuyu), Potassium hydroxide (KOH, Aladdin, 99.99%), Ruthenium-carbon (Ru/C, Aladdin, 5.0 wt.%), Platinum/carbon commercial catalyst (Pt/C, Johnson Matthey PLC, London, UK, 20 wt.%), Nafion (Sigma-Aldrich Co. LLC, St. Louis, MO, USA, 5.0 wt.%). All the chemicals were used as received without further purification, and all solutions were freshly prepared with ultrapure water. H_5_[PV_2_Mo_10_O_40_]·34H_2_O, referred to as V_2_Mo_10_, was synthesized according to the established literature methods [26].

### 2.2. Materials Preparation

#### 2.2.1. Synthesis of the V_2_Mo_10_-Based Hollow Assembly

In a typical synthesis, 0.039 g of 4,4′-bipyridine was dissolved in 25 mL of ethanol under stirring at room temperature (Solution 1). A total of 0.118 g of V_2_Mo_10_ was dissolved in a mixed solvent of 5 mL deionized water and 20 mL ethanol (Solution 2). Solution 1 was then added dropwise to Solution 2 under continuous stirring. The mixture was stirred at room temperature for 5 h, producing an orange-yellow precipitate, which was collected by centrifugation, washed with ethanol and deionized water, and dried at 60 °C overnight. Then, the final orange-yellow sample (named as V_2_Mo_10_-based hollow assembly) was obtained with a yield of 0.093 g.

A series of control samples were prepared by changing reaction time and the ethanol-to-deionized water volume ratio. The bulk V_2_Mo_10_-based assembly was obtained with the ethanol-to-deionized water volume ratio of 1:9. For comparison, PMo_12_-based assembly was synthesized under identical conditions by replacing V_2_Mo_10_ with PMo_12_.

#### 2.2.2. Synthesis of Hollow V-MoP

0.1 g of the dried V_2_Mo_10_-based hollow assembly and 1 g of NaH_2_PO_2_ were placed separately in the downstream and upstream regions of a quartz tube. Prior to phosphidation, the system was purged with N_2_ for 30 min to remove air, then calcined at 800 °C for 3 h (heating rate 5 °C min^−1^) in nitrogen. The resulting black solid was denoted as V-MoP, with a yield of 0.060 g.

To study the effects of calcination temperature and time, V-MoP-750 and V-MoP-850 were prepared at 750 °C and 850 °C, respectively, while V-MoP-1 and V-MoP-5 were obtained by varying calcination time to 1 h and 5 h.

#### 2.2.3. Synthesis of the Bulk V-MoP and Bulk MoP

Bulk V-MoP and bulk MoP were synthesized under identical conditions to hollow V-MoP, by using bulk V_2_Mo_10_-based assembly and PMo_12_-based assembly as precursors.

### 2.3. Material Characterizations

Powder X-ray diffraction (XRD) measurements were performed using a Bruker D8 diffractometer (Bruker AXS, Karlsruhe, Germany). Scanning electron microscope (SEM) testing was performed on a Hitachi S-4800 instrument (Hitachi High-Tech, Tokyo, Japan). The microstructural characteristics and elemental composition of the synthesized materials were investigated using transmission electron microscopy (TEM) coupled with energy-dispersive X-ray spectroscopy (EDX) on a JEOL JEM-2100 instrument (JEOL, Tokyo, Japan) operated at 200 kV. The X-ray photoelectron spectroscopy (XPS) analysis was carried out on Thermo Fisher Scientific ESCALAB Xi+ spectrometer (Thermo Fisher Scientific Inc., Waltham, MA, USA). The analysis chamber maintained a vacuum level of 5 × 10^−10^ Pa, with Al Kα radiation (1486.68 eV) as the excitation source. Inductively coupled plasma-atomic omission spectrometry (ICP-OES) was calculated using the PerkinElmer Optima 7000DV (PerkinElmer, Waltham, MA, USA). All samples to be tested were dissolved in HF solution. The Fourier transform infrared (FT-IR) spectra in the 400–4000 cm^−1^ region were recorded using the PE Spectrum One B infrared spectrometer with KBr pellets (PerkinElmer Inc., Waltham, MA, USA). Scanning Kelvin probe (SKP) measurement (SKP5050 system, KP Technology Ltd., Wick, Scotland, UK) was performed in ambient atmosphere with the use of a gold electrode as the reference electrode.

### 2.4. Electrochemical Measurements

Electrochemical tests were conducted at room temperature on a CHI760E electrochemical workstation (CH Instruments, Inc., Shanghai, China) using a three-electrode setup. A standard Hg/HgO electrode and graphite rod were used as the reference electrode and counter electrode. A mixture of 2.5 mg catalyst and 0.5 mg carbon black was dispersed in a solution containing 5% Nafion, DI water, and ethanol. The prepared dispersion was applied to nickel foam (NF) (1 × 1 cm) as the working electrode. Cyclic voltammetry (CV) measurements were performed in the potential ranges of −0.8 to −1.5 V (vs. Hg/HgO) for HER. Linear sweep voltammetry (LSV) was carried out at 5 mV s^−1^ under the same potential window as CV tests. A 90% iR compensation was employed for LSV. All potentials were converted to the reversible hydrogen electrode (RHE) according to E_RHE_ = E_Hg/HgO_ + 0.0977 + 0.059 pH. After CV was tested at the varied scan rates (10–60 mV s^−1^) in the non-Faradaic potential windows, electrochemical double layer capacitance (C_dl_) was calculated. Electrochemical active surface area (ECSA) values were calculated from the relationship: ECSA = specific capacitance/40 μF cm^−2^. Electrochemical impedance spectroscopy (EIS) data were gathered in the frequency range of 0.01–100,000 Hz.

## 3. Results and Discussion

### 3.1. Fabrication and Characterization

**Figure 1:**
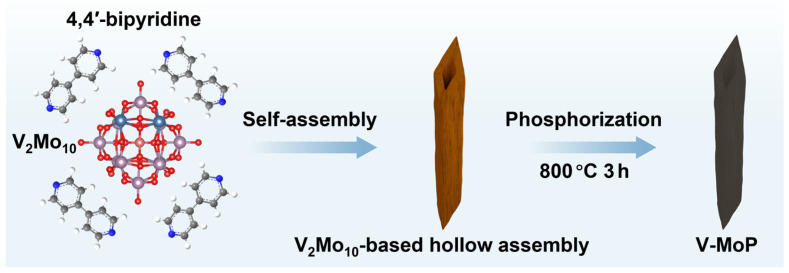
Schematic diagram of the preparation of V-MoP.

The preparation process of V-MoP is schematically illustrated in Figure 1. Initially, we designed and utilized 4,4′-bipyridine to guide the self-assembly of V_2_Mo_10_, thereby constructing a POM-based hollow tubular structure (referred to as V_2_Mo_10_-based hollow assembly). Finally, the as-prepared V_2_Mo_10_-based hollow assembly precursor was phosphorized at 800 °C for 3 h to yield the hollow tubular V-MoP catalyst. In brief, POMs, as a class of important metal oxide clusters, have been extensively utilized as transferable building blocks for constructing functional materials, due to their nanoscale dimensions, tunable composition and structure, and strong coordination ability [27,28]. The POM clusters with abundant terminal and bridging oxygen atoms offer diverse coordination modes to connect with transition metal ions and/or organic ligands (such as dopamine, melamine, and pyrrole), thus constructing novel and specific architectures [29,30]. For example, Zheng et al. reported a POM-based inorganic–organic hybrid supramolecular nanotube crystalline material [31]. A V-substituted Keggin structure POM (V_2_Mo_10_), in which each [VO_6_] unit is edge-shared with four [MoO_6_] octahedrons, was employed to introduce both V and Mo, thereby inducing V doping in MoP. Based on these structural characteristics, a hollow tubular Mo-based precursor (V_2_Mo_10_-based hollow assembly) was successfully synthesized via the assembly of V_2_Mo_10_ and the rigid 4,4′-bipyridine ligand through hydrogen bonding and electrostatic interaction. The monitored color change during the experiment indicates the successful formation of a new assembly (Figure S1). The SEM images (Figure S2) reveal that the V_2_Mo_10_-based hollow assembly precursor possesses an open-ended hollow tubular structure with an average diameter of approximately 2.5 μm and an outer wall thickness of approximately 150 nm. Furthermore, the surfaces of the V_2_Mo_10_-based hollow assembly precursor are relatively smooth, devoid of obvious defects or irregularities. XRD and FT-IR characterizations further confirm the successful synthesis of the precursor (Figures S3 and S4).

To elucidate the formation mechanism of the hollow tubular structure, a series of control experiments were carried out by varying the ethanol-to-water ratio and reaction time. When the ethanol ratio was low, a bulky, irregular morphology is obtained (Figure S5a). As the ethanol ratio increases, the sample gradually evolves into partially hollow tubular structures (Figure S5b). A further increase in ethanol ratio produces the open-ended hollow tubular structure V_2_Mo_10_-based hollow assembly. Given that the ethanol-to-water ratio directly determines the polarity of the system, precise adjustment of polarity controls the nucleation and growth rates of the precursors. Additionally, a systematic investigation into the reaction time elucidates the kinetic pathway of this morphology evolution, corroborating the dominance of the Ostwald ripening mechanism. When the reaction time was short, the as-prepared precursor exhibited a solid structure with no hollow features (Figure S6a). As the stirring time was prolonged, the continuous ripening process induces the formation of hollow tubular structures (Figure S2). However, further extending the reaction time led to the collapse of these structures, accompanied by a significant increase in particle size (Figure S6b). Thus, an assembly process based on the V_2_Mo_10_ POM undergoes an Ostwald ripening mechanism, during which polarity and reaction time jointly determine the final morphology. Furthermore, when V_2_Mo_10_ was replaced by PMo_12_, a large, irregular block-like structure (Figure S7) was formed. The significant change in the morphology is likely attributed to the lower negative charge and higher acidity of PMo_12_, which is not conducive to forming hollow tubular morphology, highlighting the key role of V_2_Mo_10_ in achieving the desired hollow tubular assembly. Furthermore, in contrast to conventional hard-template methods that often suffer from structural collapse and cumbersome template removal [32,33], this work utilizes V-substituted Mo-based POM precursors to fabricate V-doped hollow MoP in a template-free manner, wherein the atomic precision of POM ensures accurate V incorporation.

**Figure 2:**
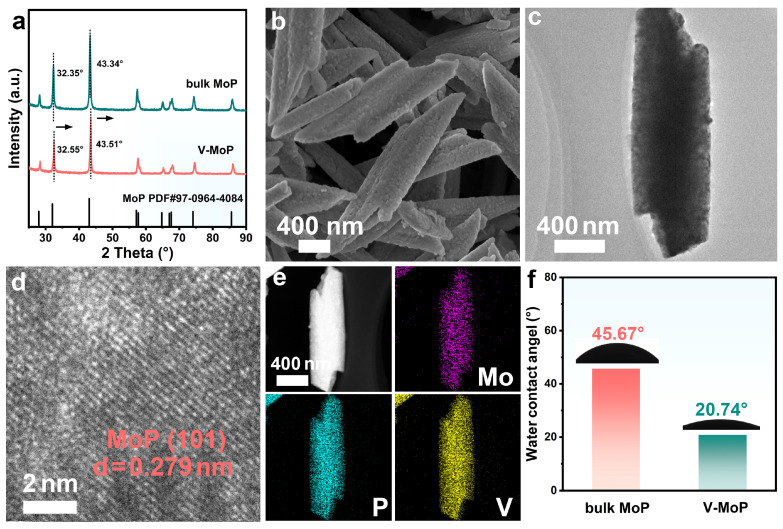
(**a**) XRD patterns of bulk MoP and V-MoP. (**b**) SEM image and (**c**) TEM image of V-MoP. (**d**) HRTEM image of V-MoP. (**e**) STEM image and EDS elemental mappings of V-MoP. (**f**) Water contact angles of bulk MoP and V-MoP.

To analyze the crystal structure of materials, XRD measurements were carried out. As shown in Figure 2a, V-MoP exhibits sharp diffraction peaks characteristic of the hexagonal phase of MoP (JCPDS No. 97-064-4084), but no diffraction peaks related to V species are observed. Notably, compared to bulk MoP, the diffraction peaks of V-MoP exhibit a systematic shift toward higher angles, suggesting a slight contraction in the interplanar spacing, which can be attributed to the replacement of Mo atoms (r_Mo_ = 139 pm) by smaller-radius V atoms (r_V_ = 134 pm). The above results indicate that V atoms have been successfully doped into MoP [34,35]. Inductively coupled plasma (ICP-OES) measurements confirm that the V content in V-MoP is only 1.3 wt.%. The microstructure of the V-MoP catalyst was characterized using SEM and transmission electron microscopy (TEM). As shown in Figure 2b,c, V-MoP also shows a unique hollow tubular morphology with the average diameter of approximately 2 μm. Meanwhile, significant shrinkage was observed, which can be attributed to the decomposition of organic components and volume contraction during the high-temperature calcination process. Notably, distinct shell pores appeared on the outer wall of the V-MoP hollow tubes, providing more exposed surface area for active sites, promoting mass transfer efficiency, and thereby significantly enhancing HER performance. The high-resolution TEM (HRTEM) image (Figure 2d) reveals lattice fringes with an interplanar spacing of 0.279 nm, which is attributed to the (101) plane of MoP. In addition, the corresponding energy-dispersive spectroscopy (EDS) elemental mapping images (Figure 2e and Figure S8) confirm that V, Mo, P, C and N are uniformly distributed throughout the hollow tube structure. The C and N components originate from the thermal decomposition of 4,4′-bipyridine ligands, which potentially enhances electrical conductivity. To further verify the pore structure characteristics of V-MoP, nitrogen adsorption and desorption tests were conducted. The Brunauer–Emmett–Teller (BET) specific surface area (S_BET_) of V-MoP is 22.9 m^2^ g^−1^ (Figure S9), which is significantly higher than the S_BET_ values of bulk MoP (4.3 m^2^ g^−1^) and bulk V-MoP (9.4 m^2^ g^−1^). It is worth noting that V-MoP hollow tubular has a larger pore volume (Figure S10), which facilitates electrolyte penetration and gas diffusion at the solid–liquid interface, while also exposing more active sites, thereby promoting the electrocatalytic reaction process. Simultaneously, the hollow tubes with porous outer walls serve as highly efficient transport channels, promoting rapid electrolyte wetting while facilitating the timely desorption of hydrogen bubbles, thereby significantly reducing mass transfer resistance during the HER [36]. Furthermore, the increased surface roughness of the hollow tubes synergistically improves the material’s surface hydrophilicity, allowing the electrolyte to spread uniformly across the electrode interface. To investigate the hydrophilicity, water contact angle measurements were conducted (Figure 2f). V-MoP shows a significantly lower contact angle (20.74°) than that of bulk MoP (45.67°), suggesting that V-MoP has good wettability, enhancing mass transfer efficiency. This difference indicates that the open, hollow structure facilitates fluid transport and gas release, while V doping alters the surface properties of MoP, enhancing its inherent hydrophilicity. The synergistic effect of these two factors thus enables more efficient mass transfer.

**Figure 3:**
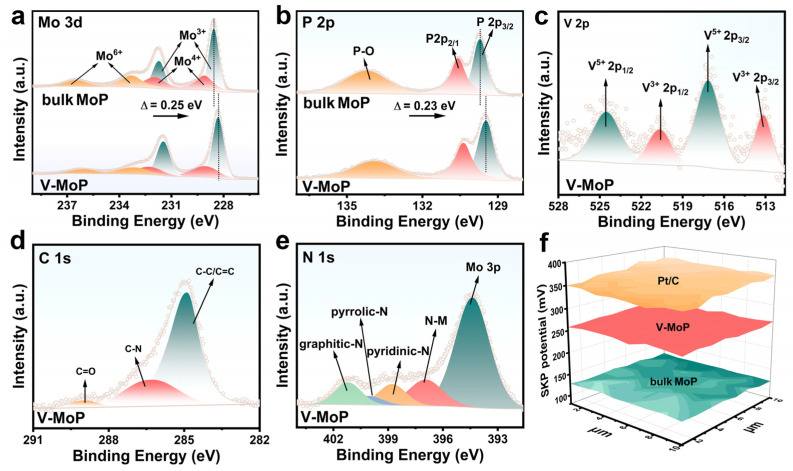
(**a**) Mo 3d and (**b**) P 2p XPS spectra of bulk MoP and V-MoP. (**c**) V 2p, (**d**) C1s and (**e**) N1s XPS spectra of V-MoP. (**f**) WF drawings of V-MoP, bulk MoP and Pt/C.

To determine the chemical composition and valence states of the prepared materials, V-MoP and bulk MoP were investigated by X-ray photoelectron spectroscopy (XPS). The survey spectrum of V-MoP shows the coexistence of V, Mo, P, C, N, and O elements, confirming the successful incorporation of V atoms (Figure S11). In the Mo 3d spectrum of V-MoP (Figure 3a), the peaks located at 228.31 and 231.47 eV correspond to Mo 3d_5/2_ and Mo 3d_3/2_ of Mo^3+^ in Mo-P bonds, respectively [37]. The Mo^3+^ species is highly active for the HER, considering its vacant energy levels facilitate water adsorption, thereby promoting water dissociation [38]. The presence of Mo^4+^ (229.06/232.28 eV) and Mo^6+^ (233.14/236.11 eV) is assigned to the susceptibility of the Mo-terminated surface to air oxidation [39]. Remarkably, binding energies of the Mo peaks in V-MoP is 0.25 eV lower than that in bulk MoP, suggesting that V incorporation modulates the electronic environment of Mo. As illustrated in Figure 3b, the fitted peaks at 129.46 eV and 130.36 eV can be attributed to the P 2p_3/2_ and P 2p_1/2_ energy levels of phosphorus–metal (P–M) bonding, respectively, while the peak at 133.85 eV indicates the presence of P–O bonds [40]. Similarly, the P–M peaks of V-MoP exhibit a slight negative shift, suggesting an increase in the average electron density around the P sites after V doping. As shown in Figure 3c, the V 2p XPS spectrum displays two pairs of spin–orbit doublets at 513.16/520.67 eV and 517.15/524.52 eV, corresponding to V^3+^ and V^5+^, respectively, confirming the successful incorporation of V into the matrix [41,42]. The incorporation of V modulates the electronic structure of MoP, leading to electron enrichment at the Mo and P sites. The presence of mixed-valence V species is expected to improve the catalytic activity of MoP [43]. In the C 1s spectrum of V-MoP (Figure 3d), the three distinct peaks correspond to C=O (288.98 eV), C–N (286.26 eV), C–C/C=C (284.89 eV) [44]. The high proportion of sp^2^-hybridized carbon underscores the pivotal role of graphitic carbon, which contributes to enhanced electronic conductivity. Furthermore, the N 1s XPS spectrum (Figure 3e) confirms the presence of graphitic N (401.19 eV), pyrrolic N (400.06 eV), pyridinic N (398.78 eV) and M–N bonds (396.94 eV), indicating that N has been successfully doped into the carbon layer [45]. As described in the report, the M-N bonds facilitate charge transfer [46]. The above results indicate that V doping causes electron enrichment at the Mo and P sites, which is expected to optimize the adsorption behavior of reaction intermediates, thereby significantly improving HER activity. Given that the work function (WF) characterizes the minimum energy required for electrons to escape from a material’s surface, scanning Kelvin probe (SKP) measurements were conducted to determine the WF and evaluate charge transfer dynamics during electrocatalysis. Pt readily captures electrons, owing to its high work function [47]. During the HER process, electrons are required for adsorbed H* to generate hydrogen. Therefore, if a catalyst possesses a high WF value that approaches or exceeds that of Pt, it is likely to exhibit high activity [48]. As shown in Figure 3f, V-MoP exhibits a work function value closer to that of Pt/C. This suggests that the introduction of V effectively optimizes the material’s electronic structure, endowing it with charge capture capabilities comparable to those of Pt, thereby significantly enhancing HER activity [49,50].

### 3.2. Electrocatalytic Performance

**Figure 4:**
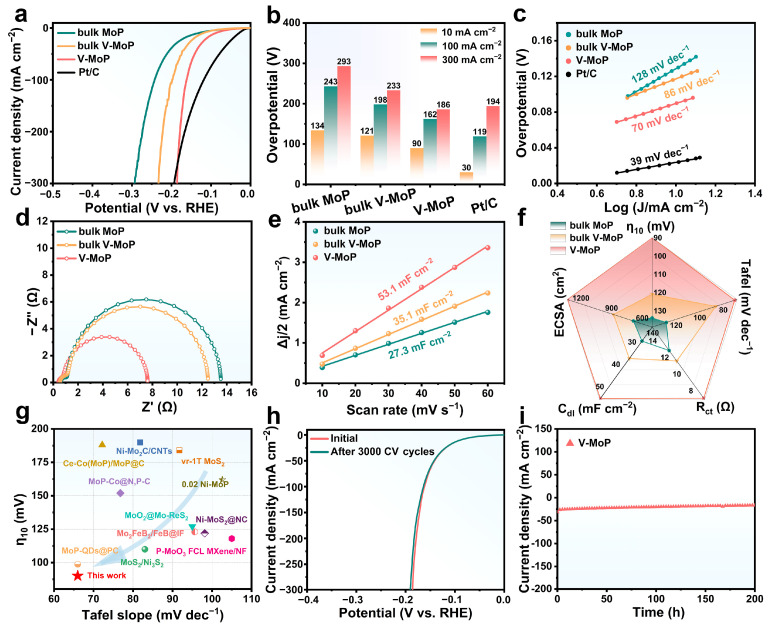
(**a**) HER polarization curves in 1.0 M KOH. (**b**) Corresponding overpotentials (η) at different current densities. (**c**) Tafel plots. (**d**) Nyquist plots. (**e**) Capacitive currents as a function of the scan rate. (**f**) Comparisons of HER performance in terms of η_10_, Tafel slope, R_ct_ value, C_dl_, and ECSA. (**g**) Comparison of η_10_ and Tafel slope with those of reported Mo-based electrocatalysts. (**h**) Polarization curves of V-MoP before and after 3000 cycles of CV. (**i**) Chronopotentiometric curve of V-MoP for 200 h.

The HER activity of V-MoP was evaluated in 1.0 M KOH by utilizing a standard three-electrode system. To avoid any possible effect of Pt, a graphite rod was used as counter electrode. To investigate the influence of different reaction conditions on the catalytic activity, a comparative analysis was performed on control samples prepared at different phosphidation temperatures and for different phosphidation times (Figure S12). To further investigate the role of V doping and morphological effect on the HER performance, bulk V-MoP and bulk MoP were also tested and compared. As shown in Figure 4a, linear sweep voltammetry (LSV) polarization curves with 90% iR compensation reveal that the optimized V-MoP achieves an overpotential of 90 mV at a current density of 10 mA cm^−2^, which is significantly lower than that of bulk MoP (134 mV) and bulk V-MoP (121 mV). Furthermore, even at high current densities of 100 and 300 mA cm^−2^, V-MoP maintains lower overpotentials (162 and 186 mV) compared to bulk MoP (243 and 293 mV) and bulk V-MoP (198 and 233 mV) (Figure 4b). Notably, when the current density exceeds 260 mA cm^−2^, the overpotentials required by V-MoP are lower than those of commercial 20% Pt/C. The comparison between bulk MoP and bulk V-MoP reflects the contribution of V doping to HER activity. Furthermore, the performance difference between hollow V-MoP and bulk V-MoP can be mainly attributed to the hollow structural effect, which is expected to improve electrolyte accessibility and mass transport. Overall, the enhanced performance arises from the combined effects of morphology design and electronic regulation. The reaction kinetics was evaluated by Tafel analysis [51,52]. As shown in Figure 4c, the Tafel slope of V-MoP is 70 mV dec^−1^, slightly higher than that of Pt/C (39 mV dec^−1^) but significantly lower than that of MoP (128 mV dec^−1^) and bulk V-MoP (86 mV dec^−1^). This indicates that the reaction kinetics of V-MoP are improved, which is consistent with its enhanced catalytic activity. The HER on V-MoP follows the Volmer–Heyrovsky mechanism, where the Heyrovsky step acts as the rate-determining step (RDS). Furthermore, the significant reduction in the Tafel slope indicates that the unique microtubule morphology helps increase the electrochemical surface area and facilitates electron and mass transfer processes. At the same time, the introduction of V greatly accelerates the water dissociation step, highlighting the significant impact of electronic regulation on active sites.

Electrochemical impedance spectroscopy (EIS) further reveals the differences in charge transfer efficiency. The Nyquist plot (Figure 4d and Figure S13) reveals charge transfer resistances (R_ct_) of 6.4 Ω for V-MoP and 12.2 Ω for MoP, 10.9 Ω for bulk V-MoP. Compared with MoP and bulk V-MoP, V-MoP exhibits a lower charge transfer resistance, indicating the fastest charge transfer rate at the interface between V-MoP and the electrolyte. This is likely attributed to an increase in electrical conductivity resulting from the combination of morphology engineering and electronic structure modulation. To deeply investigate the exposed active sites of the V-MoP hollow tubular for alkaline HER, the double-layer capacitance (C_dl_) was tested to determine the electrochemically active surface area (ECSA). V-MoP hollow tubular shows a higher C_dl_ (53.1 mF cm^−2^) than that of bulk MoP (27.3 mF cm^−2^) and bulk V-MoP (35.1 mF cm^−2^) (Figure S14 and Figure 4e), which suggests that V-MoP hollow tubular has a largest ECSA value (Figure S15), indicating its more accessible active sites. Compared to bulk MoP, the increase in ESCA is observed in bulk V-MoP, which reflects the effect of V doping, while for V-MoP hollow tubular, the further enhancement in ESCA arises from the hollow structure. Furthermore, the ECSA-normalized LSV curves (Figure S16) show that V-MoP hollow tubular delivers highest current density at the same potential, indicating that the individual active site within the V-MoP hollow tubular possesses superior intrinsic activity. Accordingly, regarding metrics such as, η_10_, Tafel slope, ESCA, R_ct_, and C_dl_, V-MoP demonstrates significantly superior HER performance compared to MoP and bulk V-MoP (Figure 4f). Impressively, V-MoP ranks among the best HER electrocatalysts reported in terms of overpotential and Tafel slope (Figure 4g and Table S1). TOF analysis (Figure S17) further confirms its superior intrinsic activity. V-MoP hollow tubular displays a lowest TOF value of 1.14 s^−1^ compared to bulk MoP (0.20 s^−1^) and bulk V-MoP (0.26 s^−1^), respectively. This enhancement arises from the combined contributions of V doping and morphology design, which together improve active site accessibility and reaction kinetics.

The long-term operational stability is an important parameter for evaluating the practical application potential of electrocatalysts. The LSV curves of V-MoP catalyst before and after 3000 cycles of CV show negligible change, indicating that the hollow tube V-MoP exhibits excellent stability (Figure 4h). In the chronopotentiometric test, V-MoP exhibits a nearly unaltered HER performance over a period of 200 h at 10 mA cm^−2^, demonstrating its superior long-term stability (Figure 4i). To evaluate its stability, post-HER characterizations including XRD and SEM were performed. The XRD patterns show that the crystalline phase of bulk MoP remains unchanged, indicating that the crystal structure was well preserved (Figure S18). The SEM image reveals that the hollow tubular morphology is well-maintained without any noticeable structural collapse (Figure S19). Meanwhile, XPS spectra (Figure S20) reveal an increased proportion of high-valence Mo, V, and P species, indicating slight surface oxidation. These results collectively demonstrate the robust structural stability of the hollow tube V-MoP catalyst during HER.

### 3.3. Overall Water Splitting Performance

**Figure 5:**
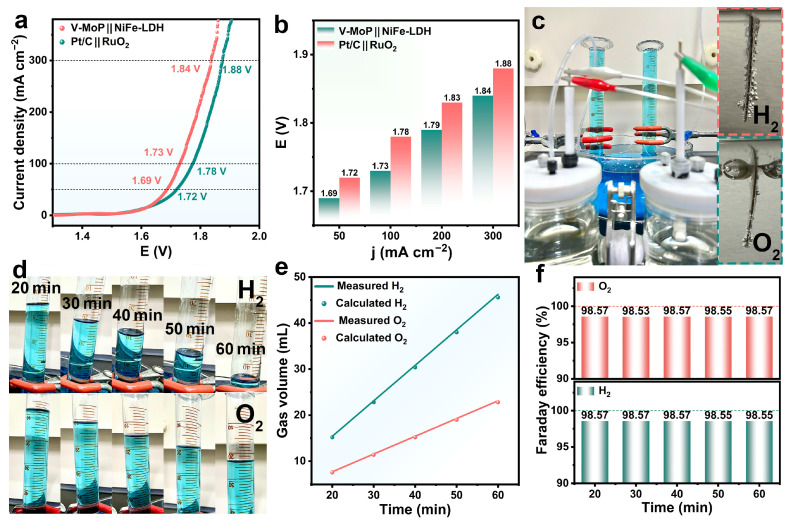
(**a**) Polarization curves. (**b**) Comparison of cell voltage at different current densities of V-MoP || NiFe-LDH and Pt/C || RuO_2_. (**c**) Digital photographs of the drainage method device of V-MoP || NiFe-LDH. (**d**) The volume of calculated and measured H_2_ and O_2_ for V-MoP || NiFe-LDH. (**e**) Faraday efficiency. (**f**) A device for collecting gas by drainage and the volume of gas produced over a period of times.

The HER, as part of the overall water splitting (OWS) process, also plays a significant role in the water electrolysis process. As shown in Figure 5a, given the excellent HER activity of V-MoP in alkaline media, a two-electrode electrolyzer (V-MoP || NiFe-LDH) was assembled using V-MoP as the cathode and NiFe-LDH as the anode in 1 M KOH electrolyte. For comparison, an electrolyzer (RuO_2_ || Pt/C) was also assembled using commercial RuO_2_ and Pt/C catalysts as the anode and cathode, respectively. V-MoP || NiFe-LDH demonstrates superior performance compared to commercial RuO_2_ || Pt/C and most reported electrocatalysts (Figure 5b and Table S2). Specifically, V-MoP || NiFe-LDH requires cell voltages of 1.69, 1.73, 1.79 and 1.84 V to reach 50, 100, 200 and 300 mA cm^−2^, respectively, which are significantly lower than those of the noble-metal-based electrolyzer (1.72, 1.78, 1.83 and 1.88 V) at the same current densities. This demonstrates that the low-cost transition metal-based V-MoP catalyst represents a cost-effective and energy-efficient approach for hydrogen production, serving as a viable alternative to commercial noble-metal catalysts. Additionally, the Faradaic efficiencies (FEs) of the V-MoP || NiFe-LDH electrode for the HER and oxygen evolution reaction (OER) were quantitatively determined via the water displacement method. The generation rates of H_2_ and O_2_ increase linearly with electrolysis time at a current density of 100 mA cm^−2^, maintaining a stoichiometric ratio of 2:1, which is consistent with the overall water splitting reaction (2H_2_O → 2H_2_ + O_2_) (Figure 5c,d). As can be clearly seen, a large number of bubbles are formed on the surfaces of both electrodes. The experimentally measured gas volumes closely match the theoretical values calculated based on the total passed charge, yielding FEs of over 98% for both the HER and OER (Figure 5e,f). Accordingly, this system demonstrates excellent compatibility with renewable energy systems and holds immense potential for green hydrogen production.

The excellent HER performance of the V-MoP hollow tube may be attributed to the following reasons: (1) The open-ended hollow tubular structure provides abundant accessible active sites, shortens the diffusion pathways of reactants, and facilitates rapid release of evolved H_2_ bubbles, thereby significantly reducing concentration polarization and ohmic polarization. (2) V incorporation, especially the coexistence of V^3+^ and V^5+^, modulates the electronic structure of Mo and P. The combination of morphology engineering and electronic structure modulation lowers the Tafel slope and accelerates the Volmer step, enabling high HER activity even at large current densities, making it a promising non-noble-metal candidate.

## 4. Conclusions

In summary, an open-porous tubular V-MoP catalyst was successfully fabricated through an assembly-induced Ostwald ripening mechanism based on the bimetallic polyoxometalate (V_2_Mo_10_), followed by subsequent phosphorization. This approach concurrently realized precise V doping within the MoP lattice and the construction of a hollow architecture with a high specific surface area. While V doping effectively optimizes the electronic structure of MoP to enhance its intrinsic activity, the open tubular morphology provides abundant accessible active sites and facilitates mass transport and gas evolution via axial channels. The resultant V-MoP achieves a 33% and 26% lower overpotential at 10 mA cm^−2^ relative to bulk MoP and bulk V-MoP, demonstrating dramatically enhanced HER activity. Notably, the water electrolysis cell assembled with V-MoP electrodes requires a lower voltage than commercial Pt/C||RuO_2_ systems, demonstrating significant potential for energy-efficient hydrogen production. This strategy provides a versatile route to design precisely metal doped, hierarchically assembled structure catalysts based on polyoxometalate, highlighting the pivotal role of morphology engineering and electronic modulation in optimizing HER performance.

## Data Availability

The original contributions presented in this study are included in the article/supplementary materials. Further inquiries can be directed to the corresponding authors.

## Supplementary Materials

The following supporting information can be downloaded at https://www.mdpi.com/article/10.3390/nano16120776/s1. Figure S1. Photographs of the synthesis process for precursor: (a) The ethanol solution of 4,4′-bipyridine. (b) The solution of V_2_Mo_10_. (c) The solution after mixing the V_2_Mo_10_ with the 4,4′-bipyridine; Figure S2. (a,b) SEM images of V_2_Mo_10_-based hollow assembly; Figure S3. XRD patterns of V_2_Mo_10_-based hollow assembly precursor, V_2_Mo_10_ and 4,4′-bipyridine; Figure S4. FT-IR spectra for V_2_Mo_10_-based hollow assembly precursor, V_2_Mo_10_ and 4,4′-bipyridine; Figure S5. SEM images of the samples synthesized with different ethanol-to-water ratios: (a) 1:9; (b) 4:1; Figure S6. SEM images of the samples synthesized with different reaction time: (a) 1 h; (b) 9 h; Figure S7. (a,b) SEM images of PMo_12_-based assembly; Figure S8. EDS elemental mappings of C and N in V-MoP; Figure S9. N2 adsorption/desorption isotherms of bulk MoP, bulk V-MoP, and V-MoP; Figure S10. BJH pore size distribution curves of bulk MoP, bulk V-MoP, and V-MoP; Figure S11. XPS survey spectrum of V-MoP; Figure S12. (a) LSV curves and (b) Tafel plots of samples obtained by varying phosphidation temperatures. (c) LSV curves and (d) Tafel plots of samples obtained by using different phosphidation times; Figure S13. Equivalent circuit used for modeling the measured electrochemical response; Figure S14. CVs of (a) bulk MoP, (b) bulk V-MoP and (c) V-MoP with different rates for HER in the region of 0.15–0.25 V; Figure S15. Comparison of the ECSA values of bulk MoP, bulk V-MoP and V-MoP for HER; Figure S16. Polarization curves normalized by ECSA of bulk MoP, bulk V-MoP and V-MoP for HER; Figure S17. CVs of (a) bulk MoP, (b) bulk V-MoP, (c) V-MoP and (d) Pt/C in 1.0 M PBS (pH = 6.87) with a scan rate of 50 mV s^−1^. (e) The calculated TOF curves for HER and (f) TOF values at overpotential of 150 mV for bulk MoP, bulk V-MoP, V-MoP and Pt/C catalysts; Figure S18. XRD patterns of V-MoP before and after stability test; Figure S19. SEM image of V-MoP after stability test; Figure S20. XPS spectra of (a) Mo 3d, (b) P 2p, (c) P 2p and (d) N 1s for V-MoP after stability test; Table S1. Comparison of HER performance of V-MoP with other Mo-based electrocatalysts in 1.0 M KOH; Table S2. Comparison of overall water splitting performance of V-MoP with recently reported Mo-based bifunctional electrocatalyst in 1 M KOH.
