# Supplementary material for: Hollow Tubular Engineering and Electronic Structure Modulation of Vanadium-Incorporated MoP for Boosting Alkaline Hydrogen Evolution
Source: Nanomaterials (Basel). 2026 Jun 19;16(12):776. doi: 10.3390/nano16120776 (PMC13305999; doi:10.3390/nano16120776)
Supplement: Supplementary file 1 [file nanomaterials-16-00776-s001.zip › nanomaterials-4357536-supplementary.pdf]

Supporting Information

Hollow Tubular Engineering and Electronic Structure Modulation of Vanadium-Incorporated MoP for Boosting Alkaline Hydrogen Evolution

Wei Yang, Guimin Wang, Siyi Yang, Ganceng Yang, Haijing Yan * and Yanqing Jiao *

Key Laboratory of Functional Inorganic Material Chemistry, National Center for International Research on Catalytic Technology, Heilongjiang University, Ministry of Education of the People's Republic of China, Harbin 150080, China

* Correspondence: yanhaijing@hlju.edu.cn (H.Y.); jiaoyanqing@hlju.edu.cn (Y.J.) or jiaoyq617@outlook.com (Y.J.)

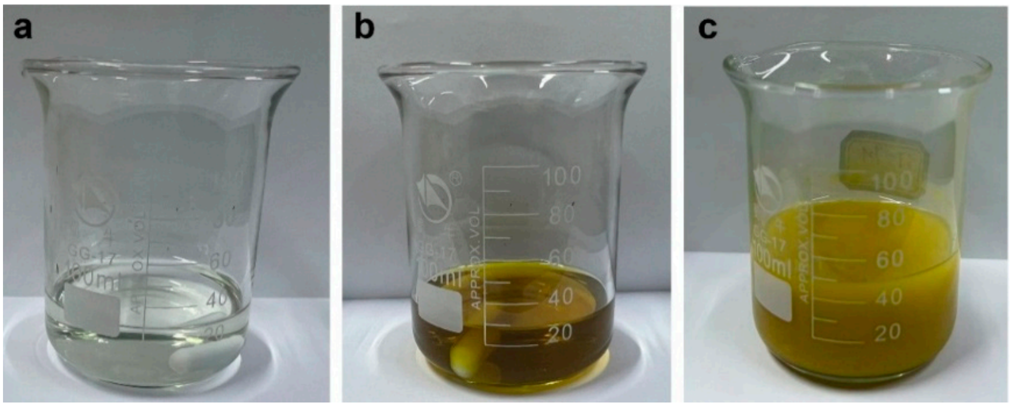

Figure S1. Photographs of the synthesis process for precursor: (a) The ethanol solution of 4,4′-bipyridine, (b) The solution of V_2Mo_{10}, (c) The solution after mixing the V_2Mo_{10} with the 4,4′-bipyridine.

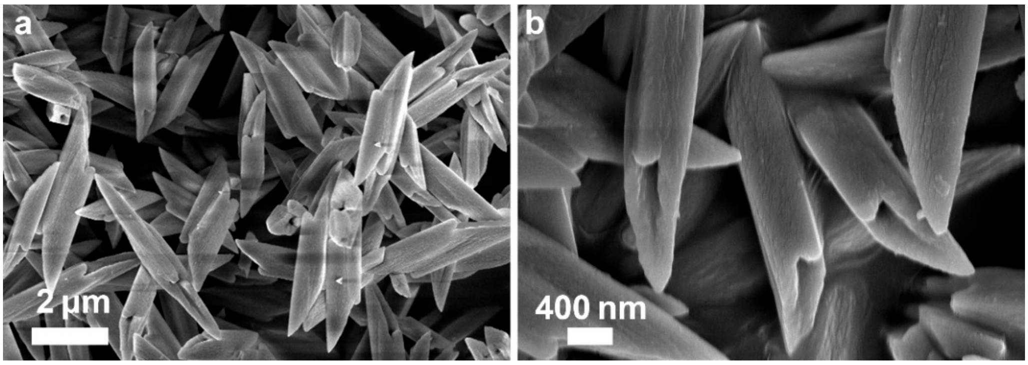

Figure S2. (a, b) SEM images of V_2Mo_{10}-based hollow assembly.

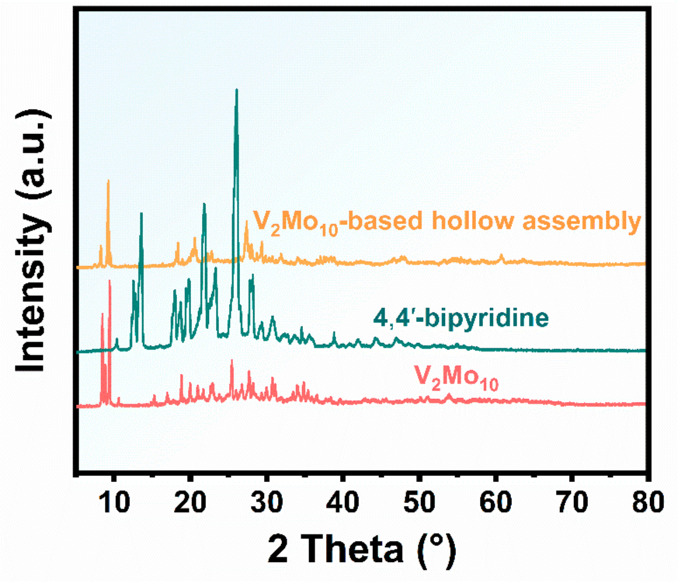

Figure S3. XRD patterns of V_2Mo_{10}-based hollow assembly, V_2Mo_{10} and 4,4′-bipyridine.

As shown in Figure S3, the XRD pattern of the precursor indicates that the diffraction peak positions and intensities of V_2Mo_{10}-based hollow assembly are significantly different from those of individual V_2Mo_{10} and 4,4′-bipyridine. This result confirms the chemical assembly between V_2Mo_{10} and 4,4′-bipyridine, leading to the formation of a composite with novel structural features. Additionally, the XRD pattern of the V_2Mo_{10}-based hollow assembly precursor displays sharp characteristic diffraction peaks, indicating that the as-prepared material possesses high crystallinity rather than an amorphous structure.

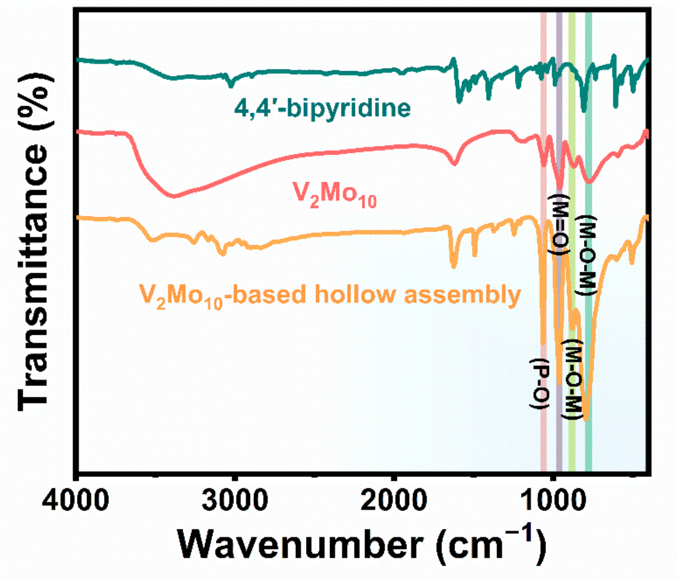

Figure S4. FT-IR spectra for V_2Mo_{10}-based hollow assembly, V_2Mo_{10} and 4,4′-bipyridine.

FT-IR spectroscopy was employed to elucidate the chemical structure of the V_2Mo_{10}-based hollow assembly precursor. The spectrum (Figure S4) displays characteristic absorption peaks at 1062, 956, 886, and 785 cm^{-1}, corresponding to the P–O, M=O, asymmetric M–O–M, and M–O–M (where M = V or Mo), bridging stretching vibrations inherent to the V_2Mo_{10} polyoxometalate framework.[1] The presence of these signature bands substantiates the successful integration of V_2Mo_{10} with 4,4′-bipyridine.

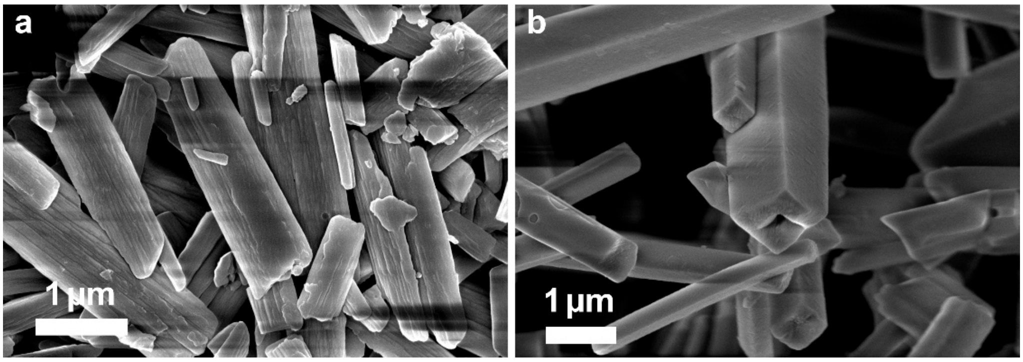

Figure S5. SEM images of the samples synthesized with different ethanol-to-water ratios:(a) 1:9; (b) 4:1.

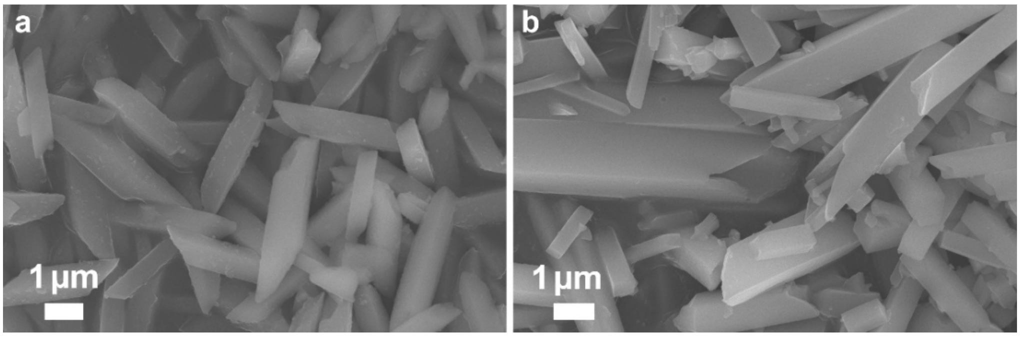

Figure S6. SEM images of the samples synthesized with different reaction time: (a) 1 h; (b) 9 h.

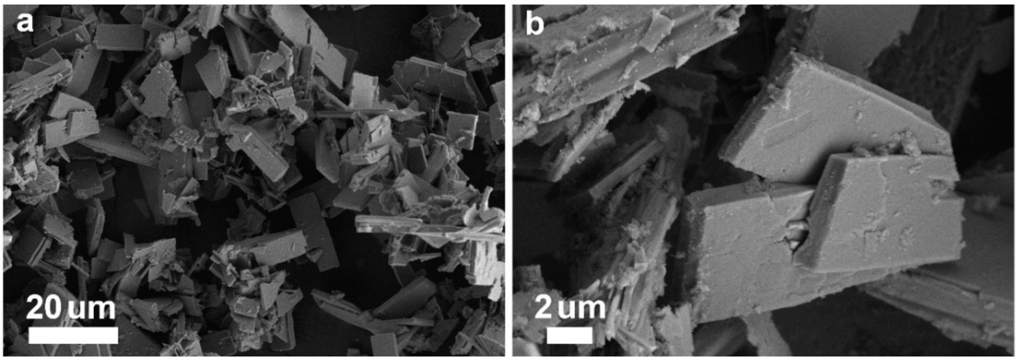

Figure S7. (a, b) SEM images of PMo_{12}-based assembly.

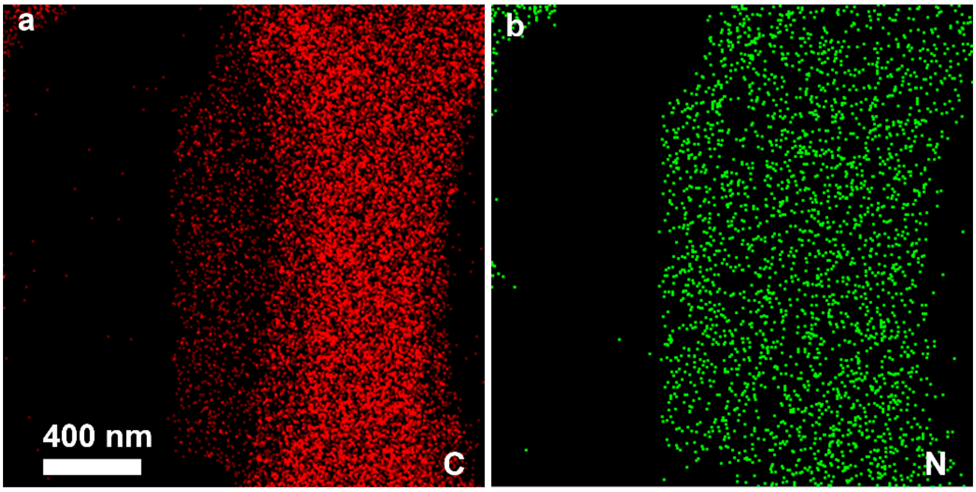

Figure S8. EDS elemental mappings of C and N in V-MoP.

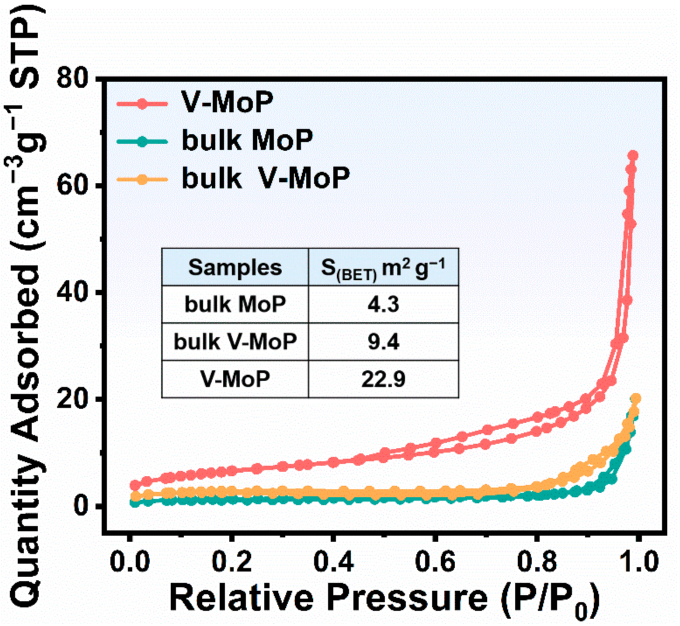

Figure S9. N_2 adsorption/desorption isotherms of bulk MoP, bulk V-MoP, and V-MoP.

.

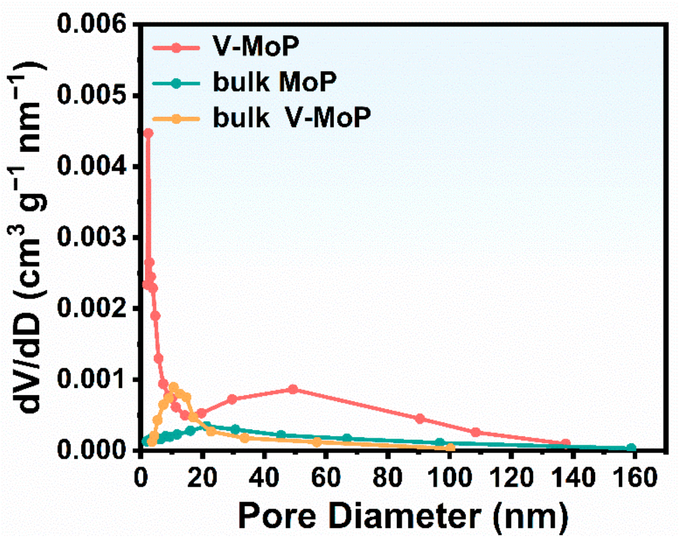

Figure S10. BJH pore size distribution curves of bulk MoP, bulk V-MoP, and V-MoP.

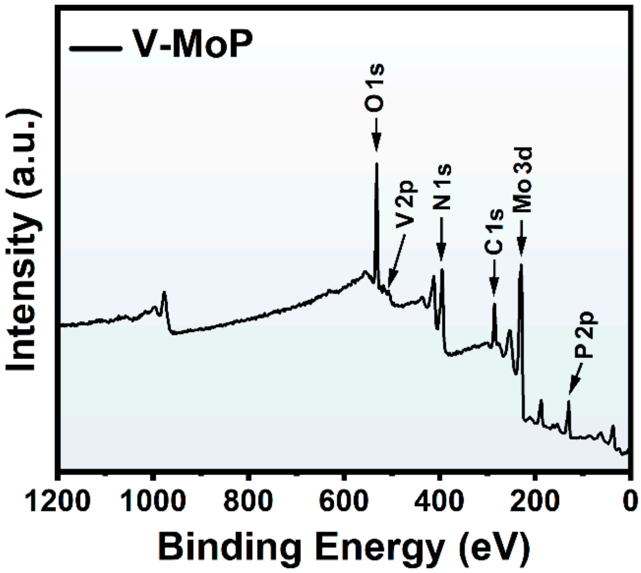

Figure S11. XPS survey spectrum of V-MoP..

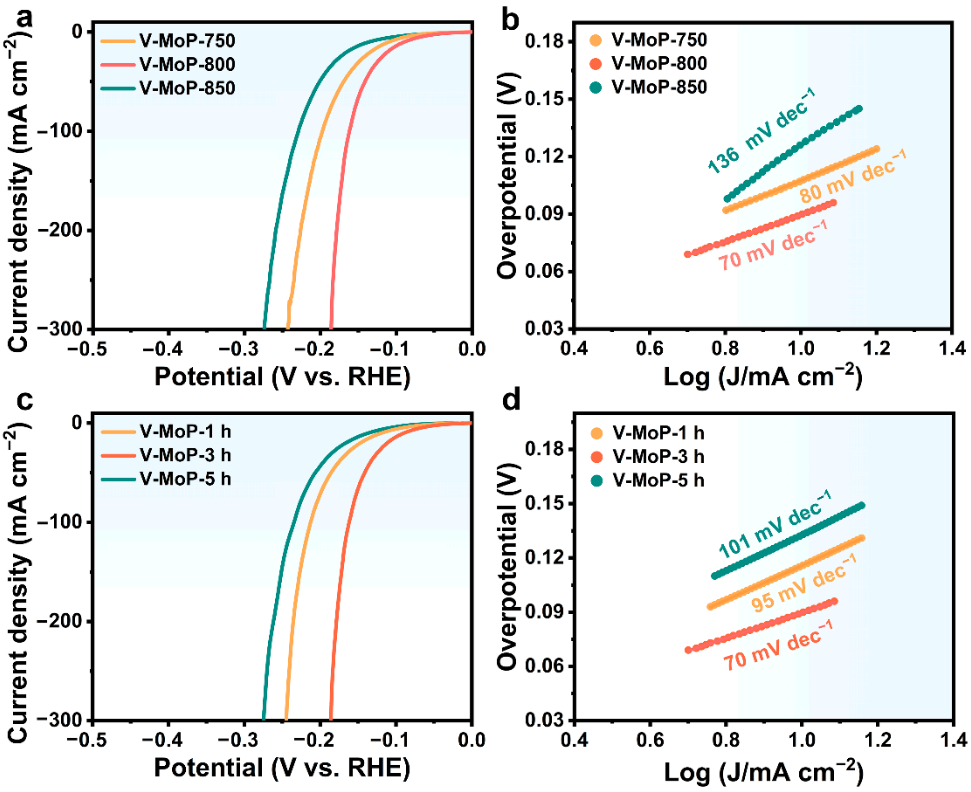

Figure S12. (a) LSV curves and (b) Tafel plots of samples obtained by varying phosphidation temperatures. (c) LSV curves and (d) Tafel plots of samples obtained by using different phosphidation times.

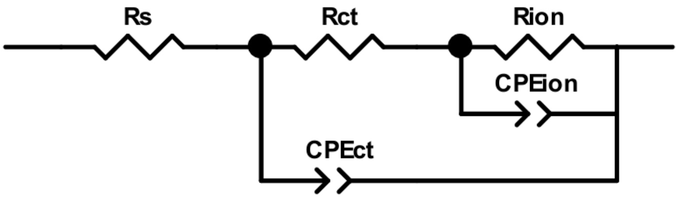

Figure S13. Equivalent circuit used for modeling the measured electrochemical response.

The model consists of two parts: the first part is R_s, representing the electrolyte resistance; the second part relates to reactions at the electrode-electrolyte interface, one of which comprises CPE_{ct} and R_{ct}, referring to the double-layer capacitance and the charge transfer resistance during the Faradaic reaction. The other set corresponds to the adsorption behavior of intermediates at the electrode-electrolyte interface and includes CPE_{ion} and R_{ion}, which represent the pseudocapacitance and ion transfer resistance generated during the adsorption/desorption of intermediates.

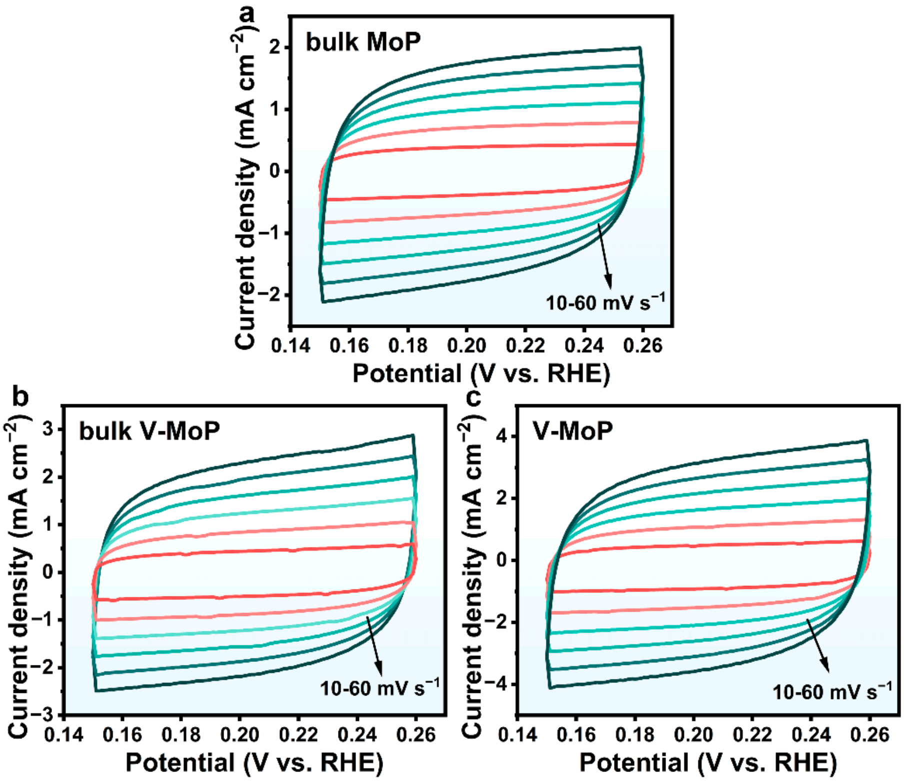

Figure S14. CVs of (a) bulk MoP, (b) bulk V-MoP and (c) V-MoP with different rates for HER in the region of 0.15-0.25 V..

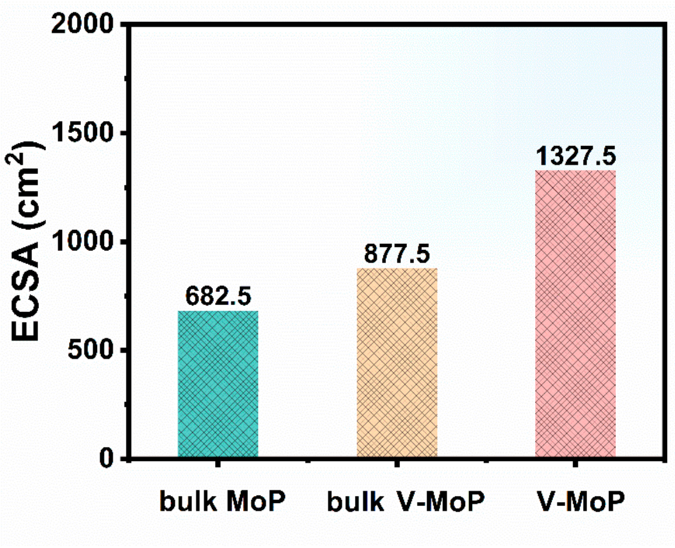

Figure S15. Comparison of the ECSA values of bulk MoP, bulk V-MoP and V-MoP for HER.

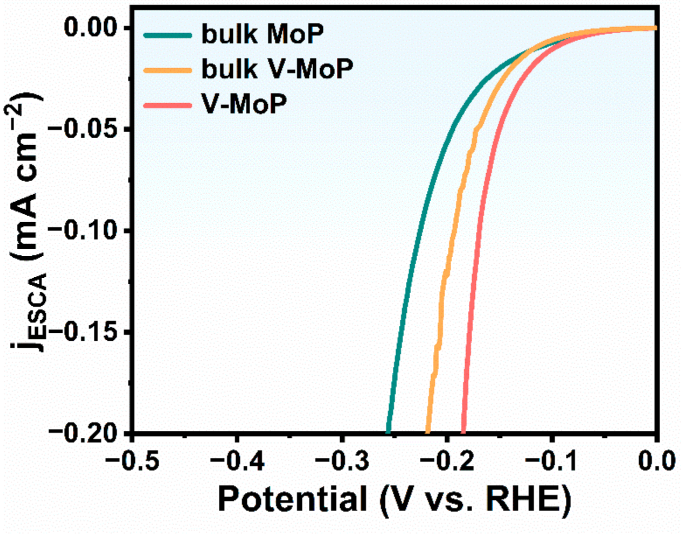

Figure S16. Polarization curves normalized by ECSA of bulk MoP, bulk V-MoP and V-MoP for HER.

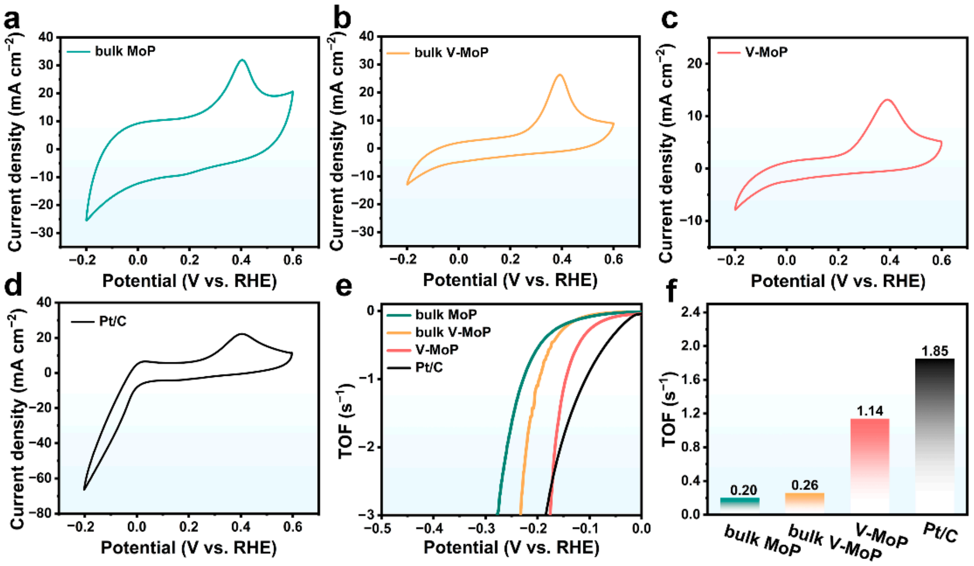

Figure S17. CVs of (a) bulk MoP, (b) bulk V-MoP, (c) V-MoP and (d) Pt/C in 1.0 M PBS (pH = 6.87) with a scan rate of 50 mV s^{-1}. (e) The calculated TOF curves for HER and (f) TOF values at overpotential of 150mV for bulk MoP, bulk V-MoP, V-MoP and Pt/C catalysts.

When the number of active sites is determined from integrating the charge amount Q of the metal redox peaks over the CV curves, the TOF (s^{-1}) can be calculated using the following equation:

$$TOF = (J \times N_A)/(\Gamma \times n \times F)$$

Where J stands for the HER current density (A cm^{-2}) in the LSV curves, N_A is the Avogadro number (mol^{-1}), F is the Faraday constant (C mol^{-1}), n is the number of electrons (for H_2, it is 2), and Γ is the number of active sites in different samples. In addition, the calculation details of Γ are as follows:

$$Q = \int d(J_{CV} \times t) = \int d(J_{CV} \times U_{CV}/v) = 1/v \int d(J_{CV} \times U_{CV}) = S/v$$

$$\Gamma = Q/e = S/(v \times e)$$

Because the $F = e \times N_A$, therefore$TOF = J \times N_A/[n \times e \times N_A \times S/(v \times e)] = (J \times v)/(n \times S)$ (s^{-1}) Where J_{CV} and U_{CV} are the current density (A cm^{-2}) and potential (V) obtained from the CV curve, ν (V s^{-1}) is the scan rate, S (V A cm^{-2}) is the integrated area of the redox peak from CV curve, and e is the elementary charge, $e = 1.602 \times 10^{-19}$ C.

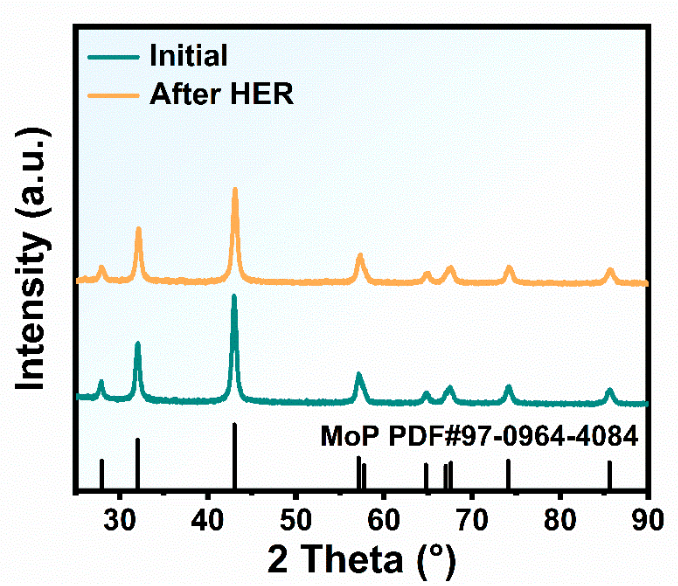

Figure S18. XRD patterns of V-MoP before and after stability test.

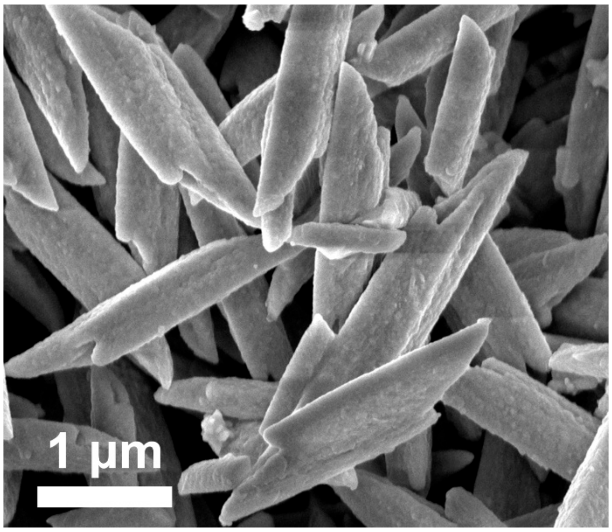

Figure S19. SEM image of V-MoP after stability test.

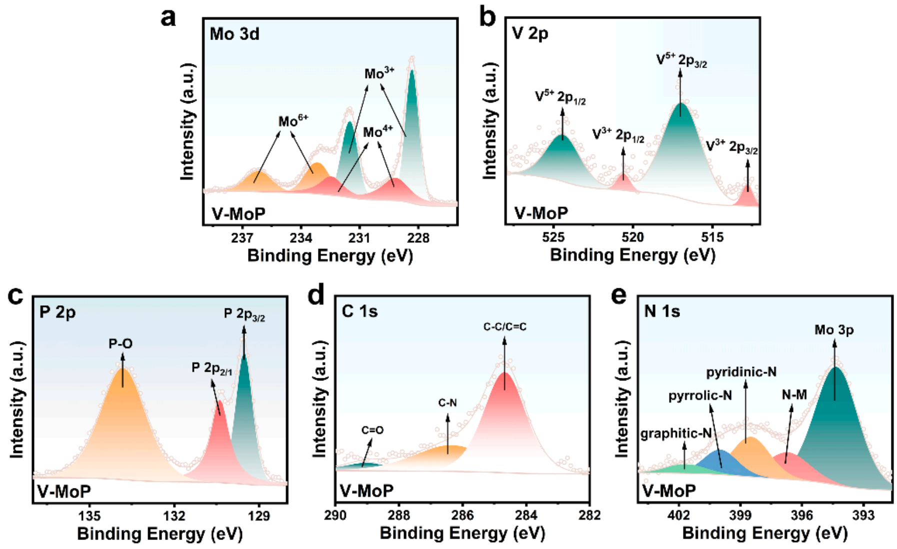

Figure S20. XPS spectra of (a) Mo 3d, (b) V 2p, (c) P 2p, (d) C 1s and (d) N 1s for V-MoP after stability test.

Table S1. Comparison of HER performance of V-MoP with other Mo-based electrocatalysts in 1.0 M KOH.

Catalyst	Electrode	Loading (mg/cm^2)	η_{10} (mV)	Tafel slope (mV/dec)	Reference
**V-MoP**	**NF**	**2.5**	**90**	**70**	**This work**
**bulk MoP**	**NF**	**2.5**	**134**	**86**	**This work**
**bulk V-MoP**	**NF**	**2.5**	**121**	**128**	**This work**
**Pt/C (20%)**	**NF**	**2.5 (0.5 mg$_{Pt}$ cm^{-2})**	**30**	**39**	**This work**
WC_{1-x}/Mo_2C@CNF	Carbon Nanofiber	/	97	42.9	[2]
MoP-QDs@PC	Glassy Carbon Electrode (GCE)	/	98.8	66	[3]
MoS_2/Ni_3S_2	NF	/	110	83	[4]
MoP/NF	NF	/	114	54.6	[5]
P-MoO_3 FCL MXene/NF	NF	/	118	105	[6]
Ni-MoS_2@NC	Carbon Cloth (CC)	/	122	98.15	[7]
Mo_2FeB_2/FeB@IF	NF		123	95.6	[8]
MoO_2@Mo-ReS_2	CC	/	127	95	[9]
MoP-Co@N, P-C	CC	5.0	152	76.8	[10]
0.02 Ni-MoP	GCE	/	162	102.6	[11]
vr-1T MoS_2	CC	/	184	91.73	[12]
Ce-Co(MoP)/MoP@C	CC	/	188	72.2	[13]
Ni-Mo_2C/CNTs	GCE		190	81.8	[14]
MoS_2/MoP/NC	GCE	5.0	208	62	[15]
10:MoCo-VS_2	CC	/	248	55	[16]
MCNTs@CoS_x@MoS_2	NF	/	285	76	[17]
Co-MoxN/Mo_2C	CC	/	297	120.7	[18]

Table S2. Comparison of overall water splitting performance of V-MoP with recently reported non-precious metal-based bifunctional electrocatalyst in 1 M KOH.

Catalyst	η_{10} (mV)	Reference
**V-MoP**	**1.59**	**This work**
MoO_2/NC/Pt	1.58	[19]
MoP-Co@N, P-C	1.59	[20]
NiC/Mo_2C@C	1.60	[21]
Mo_2S/$Co_{1-x}S$@C	1.60	[22]
Co/Mo_2C	1.62	[23]
MoP/NF	1.62	[24]
Mo_2C–Mo_2N	1.64	[25]
Ru-MoP-PV	1.65	[26]
MoO_x-FeCoCu	1.69	[27]
(Mo/Co) O_x-Cu@NF	1.72	[28]

References

[1] Yang H, Bai Y, Ouyang D, et al. Coupling Biomass Pretreatment for Enzymatic Hydrolysis and Direct Biomass-to-Electricity Conversion with Molybdovanadophosphoric Heteropolyacids as Anode Electron Transfer Carriers. *Journal of Energy Chemistry* **2021**, *58(2)*, 133-146.

[2] Zhang W, Yang L, Li Z, et al. Regulating Hydrogen/Oxygen Species Adsorption Via Built-in Electric Field -Driven Electron Transfer Behavior at the Heterointerface for Efficient Water Splitting. *Angewandte Chemie International Edition* **2024**, *63(16)*, e202400888.

[3] Liu Y, Yue C, Sun F, et al. Superhydrophilic Molybdenum Phosphide Quantum Dots on Porous Carbon Matrix for Boosting Hydrogen Evolution Reaction. *Chemical Engineering Journal* **2023**, *454*, 140105.

[4] Zhang J, Wang T, Pohl D, et al. Interface Engineering of MoS_2/Ni_3S_2 Heterostructures for Highly Enhanced Electrochemical Overall-Water-Splitting Activity. *Angewandte Chemie International Edition* **2016**, *128(23)*, 6814-6819.

[5] Jiang Y, Lu Y, Lin J, et al. A Hierarchical MoP Nanoflake Array Supported on Ni Foam: A Bifunctional Electrocatalyst for Overall Water Splitting. *Small Methods* **2018**, *2(5)*, 1700369.

[6] Li M, Sun R, Li Y, et al. The 3D Porous "Celosia" Heterogeneous Interface Engineering of Layered Double Hydroxide and P-Doped Molybdenum Oxide on MXene Promotes Overall Water-splitting. *Chemical Engineering Journal* **2022**, *431*, 133941.

[7] Wang X, Lu Z, Cao Y, et al. Multi-Synergy Enabling Ni-Doped MoS_2@N-Doped Carbon Composite as Versatile Catalysts toward Hydrogen Production and Photovoltaics. *Carbon* **2025**, *231*, 119724.

[8] Sun J, Zhao P, Liu C, et al. Mo-Assisted Boronized Iron Foam Self-Supported Electrode for Bifunctional HER/OER in Alkaline Water Electrolysis. *ChemCatChem* **2026**, *18(3)*, e01868.

[9] Singh M, Park J, Kim H, et al. Heterointerface-Driven Electronic Modulation in MoO_2@N/Mo-ReS_2 Hybrid for Efficient Alkaline HER, OER, and Overall Water Splitting. *Small* **2025**, *21(34)*, 2505906.

[10] Sun D, Lin S, Yu Y, et al. One-Pot Synthesis of N and P Co-Doped Carbon Layer Stabilized Cobalt-Doped MoP 3D Porous Structure for Enhanced Overall Water Splitting. *Journal of Alloys and Compounds* **2022**, *895*, 162595.

[11] Xiao W, Zhang L, Bukhvalov D, et al. Hierarchical Ultrathin Carbon Encapsulating Transition Metal Doped MoP Electrocatalysts for Efficient and pH-Universal Hydrogen Evolution Reaction. *Nano Energy* **2020**, *70*, 104445.

[12] Kim M K, Lamichhane B, Song B, et al. Enhancing Electrocatalytic Hydrogen Evolution of MoS_2 Enabled by Electrochemical Cation Implantation for Simultaneous Surface-Defect and Phase Engineering. *Applied Catalysis B: Environment and Energy* **2024**, *352*, 124037.

[13] Chen T, Fu Y, Liao W, et al. Fabrication of Cerium-Doped CoMoP/MoP@C Heterogeneous Nanorods with High Performance for Overall Water Splitting. *Energy & Fuels* **2021**, *35(17)*, 14169-14176.

[14] Wang W, Yang L, Chen J, et al. Realizing Electronic Modulation on Mo sites for Efficient Hydrogen Evolution Reaction. *Journal of Materials Chemistry A* **2020**, *8(35)*, 18180-18187.

[15] Chi J-Q, Chai Y-M, Shang X, et al. Heterointerface Engineering of Trilayer-Shelled Ultrathin MoS_2/MoP/N-Doped Carbon Hollow Nanobubbles for Efficient Hydrogen Evolution. *Journal of Materials Chemistry A* **2018**, *6(48)*, 24783-24792.

[16] Singh V K, Nakate U T, Bhuyan P, et al. Mo/Co Doped 1T-VS_2 Nanostructures as a Superior Bifunctional Electrocatalyst for Overall Water Splitting in Alkaline Media. *Journal of Materials Chemistry A* **2022**, *10(16)*, 9067-9079.

[17] Wang C, Zhang L, Xu G, et al. Construction of Unique Ternary Composite MCNTs@CoS_x@MoS_2 with Three-Dimensional

Lamellar Heterostructure as High-Performance Bifunctional Electrocatalysts for Hydrogen Evolution and Oxygen Evolution Reactions. *Chemical Engineering Journal* **2021**, *417*, 129270.

[18] Hu P, Yang F F, Yang F, et al. Bimetallic Organic Framework Derived Co-Mo_xN/Mo_2C Catalyst for HER/OER Bifunctional Electrocatalytic Reaction. *Journal of Colloid and Interface Science* **2025**, *680*, 427-436.

[19] Ma Y, Fang T, Xu Y, et al. Electrochemical Water Splitting by Mo-Based Metal-Organic Framework-Derived MoO_2/NC/Pt Nanostructures in a Wide pH Range. *ACS Applied Nano Materials* **2024**, *7(15)*, 17364-17372.

[20] Sun D, Lin S, Yu Y, et al. One-Pot synthesis of N and P Co-Doped Carbon Layer Stabilized Cobalt-Doped MoP 3D Porous Structure for Enhanced Overall Water Splitting. *Journal of Alloys and Compounds* **2022**, 895, 162595.

[21] Santhosh Kumar K, Thiruvengadam D, Rajan K, et al. Splendid Intrinsic Activity of NiC/Mo_2C@C Nanocubes for Efficient Water Oxidation. *ACS Applied Electronic Materials* **2024**, *7(1)*, 450-462.

[22] Ma M-Y, Yu H-Z, Deng L-M, et al. Interfacial Engineering of Heterostructured Carbon-Supported Molybdenum Cobalt Sulfides for Efficient Overall Water Splitting. *Tungsten* **2023**, *5(4)*, 589-597.

[23] Yuan S, Xia M, Liu Z, et al. Dual Synergistic Effects between Co and Mo_2C in Co/Mo_2C Heterostructure for Electrocatalytic Overall Water Splitting. *Chemical Engineering Journal* **2022**, 430.

[24] Jiang Y, Lu Y, Lin J, et al. A Hierarchical MoP Nanoflake Array Supported on Ni Foam: A Bifunctional Electrocatalyst for Overall Water Splitting. *Small* **2018**, *2(5)*, 1700369.

[25] Zhang Y, Guo P, Guo S, et al. Gradient Heating Epitaxial Growth Gives Well Lattice-Matched Mo_2C-Mo_2N Heterointerfaces that Boost Both Electrocatalytic Hydrogen Evolution and Water Vapor Splitting. *Angewandte Chemie International Edition* **2022**, *61(47)*, e202209703.

[26] Li Q, Luan X, Xiao Z, et al. Ultrafast Microwave Synthesis of Ru-Doped MoP with Abundant P Vacancies as the Electrocatalyst for Hydrogen Generation in a Wide pH Range. *Inorganic Chemistry* **2023**, *62(24)*, 9687-9694.

[27] Li J, Gu X, Chang J, et al. Molybdenum Oxide-Iron, Cobalt, Copper Alloy Hybrid as Efficient Bifunctional Catalyst for Alkali Water Electrolysis. *Journal of Colloid and Interface Science* **2022**, *606*, 1662-1672.

[28] Tartour A R, Sanad M M S, El-Hallag I S, et al. Novel Mixed Heterovalent (Mo/Co) $O_{(x)}$-Zerovalent Cu System as Bi-Functional Electrocatalyst for Overall Water Splitting. *Scientific Reports* **2024**, *14(1)*, 4601.
